# Understanding Health Systems: from Sierra Leone to WONCA

**DOI:** 10.1080/17571472.2016.1173428

**Published:** 2016-05-17

**Authors:** Luke Kane

**Affiliations:** ^a^ Lewisham Vocational Training Scheme, Queens Road Partnership, London, UK

## Why this matters to me

As a GP trainee, I am trying to understand and learn about the health systems in which I am building my career. Constant change and political turbulence can make understanding the NHS a challenge but I feel it is important to persevere and work to gain a good conceptual framework of the health system in which you function. Whilst working in an Ebola treatment centre in Sierra Leone, an unexpected aspect of my role was helping to develop the process of how cases of possible Ebola virus infection were identified, isolated and treated. This was a crash course in understanding how a health system functions. Attending the WONCA Europe Conference in 2015 enabled me to further my understanding about how health systems work and gave context to how such systems change when new pressures are put upon them, for example, the primary care health system in Turkey dealing with Syrian refugees. This matters to me, and should matter to all of us, because GPs are well placed to contribute both clinically and by developing and strengthening health systems in times of crisis.

## Key messages

• Primary care exists within an ever dynamic political, social and health environment.
• Understanding the health system in which your work empowers you, and therefore, your patient.
• Attending the WONCA conference provides a platform from which to expand your understanding of health systems in the context of global primary care.
• Humanitarian and medical crises, such as Ebola and Syria, require a robust response from the medical community; we are well placed as GPs to contribute both clinically and by helping to develop health systems which support primary care.

## Text

I spent the early part of last year working in an Ebola Treatment Centre in Sierra Leone. During our pre-deployment training, one of the infectious disease consultants asked me how far through my ID training I was. When I replied that I was a first-year GP trainee, his eyes widened and he made a hasty exit with a swift ‘*good luck, mate*!’. This got me thinking about the role of a primary care doctor in a time of crisis such as the Ebola epidemic. I began to worry that perhaps I would be ‘*in over my head*’ but hoped that my generalist clinical judgement, as well as the diploma in tropical medicine, and a GP’s trouble-shooting skills would see me through in what would undoubtedly be a stressful and dangerous environment.

Over a year later, reflecting on my role in the Ebola epidemic, I feel that I was able to contribute towards the efforts to treat and isolate Ebola sufferers. Being a GP trainee was an asset – not a hindrance. Working in partnership with my specialist colleagues we put together clinical management pathways for the centre’s patients, many of whom would eventually be diagnosed with Ebola but also with other health problems. The combination of working in this global health context and helping to create an Ebola health system pathway was challenging but very rewarding. Wanting to better understand how health systems work, in theory and practice, I received an RCGP bursary to help me attend the WONCA Europe conference, held in Istanbul, Turkey, in October 2015.

Learning about the structure of a health system was not part of my medical school curriculum. At the time, I was more interested in absorbing clinical knowledge but, as I progress, I see that without a thorough understanding of the system in which you and your patient inhabit, you do yourself, and therefore, your patient a disservice.

One of the best sessions at the WONCA conference was a workshop on comparative global health care systems. We split into groups and went through a series of prompts exploring various aspects of group members’ health systems. This was fascinating and I realised that by learning about the differences between the U.K. and, for example, Finland’s health system, it enables you to better understand your own health care environment. This seemed to be the theme of my experience at the WONCA conference: by highlighting differences and alternative ways of thinking, you can better understand the complex system you, and your patient, inhabit.

Sadly, before the conference started, two large bombs were detonated at a protest in Ankara which caused over a hundred deaths. One of the most engaging sessions of the conference was given by Dr Sebnem Fincanci on the ‘Istanbul Protocol’ which is a U.N. document on standards and procedures on how to identify and document symptoms of torture so that the documentation may serve as valid evidence in court. Her lecture was given a few days after she oversaw the autopsies of those killed in the Ankara attack and it was very powerful listening to a remarkable defender and promoter of human rights talk in the midst of chaos, killing and war. Moving on from this, the health and rights of refugees was frequently discussed among delegates. With over two million refugees currently in Turkey, it was eye-opening hearing about the local experience and the challenges of providing primary health care to such large numbers of people fleeing war in Syria.

Humanitarian and medical crises require a robust response from the medical community. As GPs we have the necessary clinical skills to provide primary care on the front line. Underlying these responses, there needs to be a proper knowledge of health systems so that issues can be identified and fixed. The WONCA conference was an introduction into the start of a long road to greater understanding of the medical environment we all exist in, one that I hope will make me a better doctor to my future patients.

## Conflict of interest

The Royal College of General Practitioners paid me a £500 bursary to attend the WONCA conference and asked me to write this article about my experience.

## Acknowledgement

I would like to sincerely thank the Royal College of General Practitioners for the bursary I received without which I would have been unable to attend the WONCA conference.

